# Supplementary material for: Synergistic Effects of Melatonin and Gamma-Aminobutyric Acid on Protection of Photosynthesis System in Response to Multiple Abiotic Stressors
Source: Cells. 2021 Jun 29;10(7):1631. doi: 10.3390/cells10071631 (PMC8306587; doi:10.3390/cells10071631)
Supplement: Supplementary file 1 [file cells-10-01631-s001.zip › cells-1205899-supplementary.pdf]

Supplementary Table S1. Measured parameters related to OJIP test (Kalaji 2017)

Abbreviation	Equation	Definition
F_V/F_o	$\varphi P_0/(1 - \varphi P_0)$	Maximum efficiency of the water diffusion reaction on the donor side of PSII
F_V/F_M	$TR_0/ABS = [1 - (F_0/F_M)]$	Relative maximal variable fluorescence
Ψ_0	$ET_0/TR_0 = (1 - V_J)$	The probability that a trapped exciton proceed an electron through ETC beyond Q_A^-
φE_0	$ET_0/ABS = [1 - (F_0/F_M)]\ \psi_0 = \phi P_0 \cdot \psi_0$	Quantum yield of electron transport (at *t* = 0)
φD_0	$1 - \varphi P_0 = (F_0/FM)$	Quantum yield of energy dissipation
Φ_{PAV}	$\varphi P_0 (1 - Vav) = \varphi P_0 (Sm / tF_M)$	Average quantum yield of primary photochemical reactions (from time 0 to tF_M).
ABS/RC	$M_0 (1/VJ)(1/\varphi P_0)$	Light absorbance flux for PSII antenna Chlorophylls per reaction center
TR_0/RC	$M_0 (1/V_J)$	Trapped energy flux per reaction center
ET_0/RC	$M_0 (1/V_J)\ \psi_0$	Electron transport flux per reaction center
DI_0/RC	$(ABS/RC) - (TR_0/RC)$	Energy flux not intercepted by an RC, dissipated in the form of heat, fluorescence, or transfer to other systems, at time t = 0.
PI_{abs}	$(RC/ABS) \times (\varphi P_0/(1 - \varphi P_0)) \times (\psi_0/(1 - \psi_0))$	Performance index per absorbed light

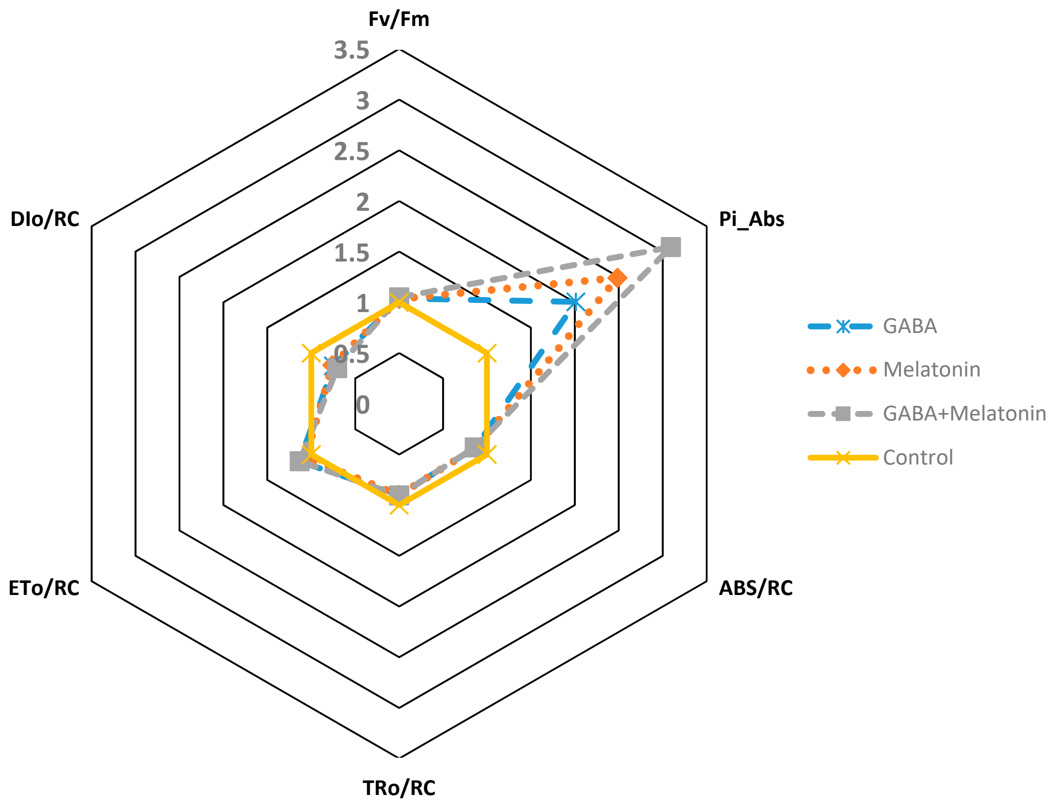

Supplementary Figure S1. The effect of Gamma-Aminobutyric Acid [(GABA (20 µM)], melatonin (200 µM) and GABA (20 µM) + melatonin (200 µM) on OJIP transients of *Vicia faba*

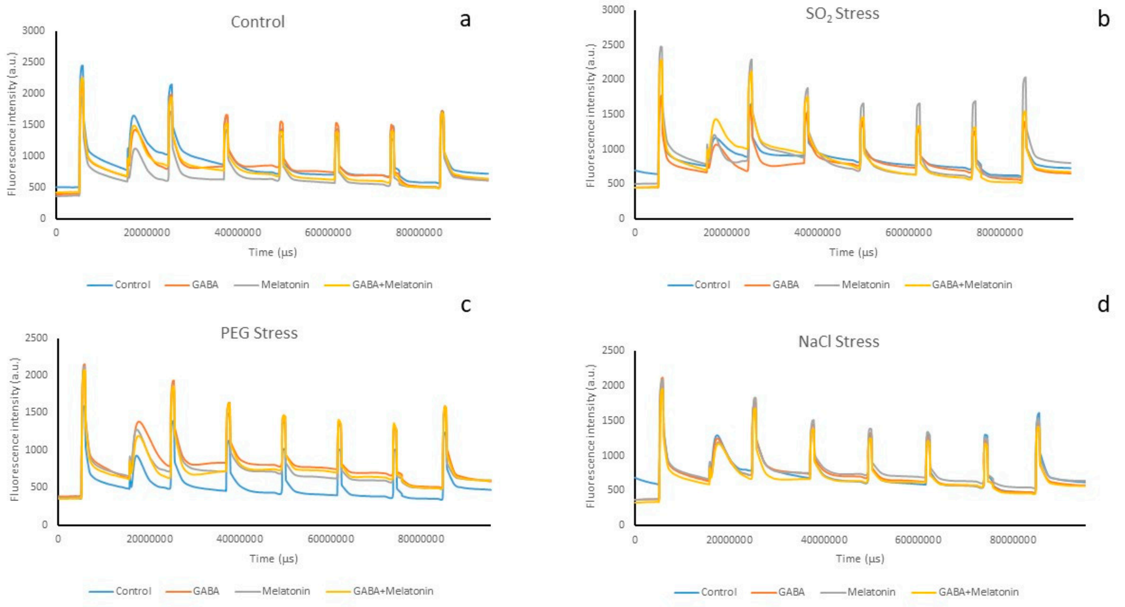

Supplementary Figure S2. Fluorescence induction kinetics of *Vicia faba* plants primed with GABA (20 µM), melatonin (200 µM) and GABA (20 µM) + melatonin (200 µM) (a), SO_2 stress (2 ppm) (b), PEG stress (-8 bar) (c) and when exposed to NaCl stress (100 mM) (d).

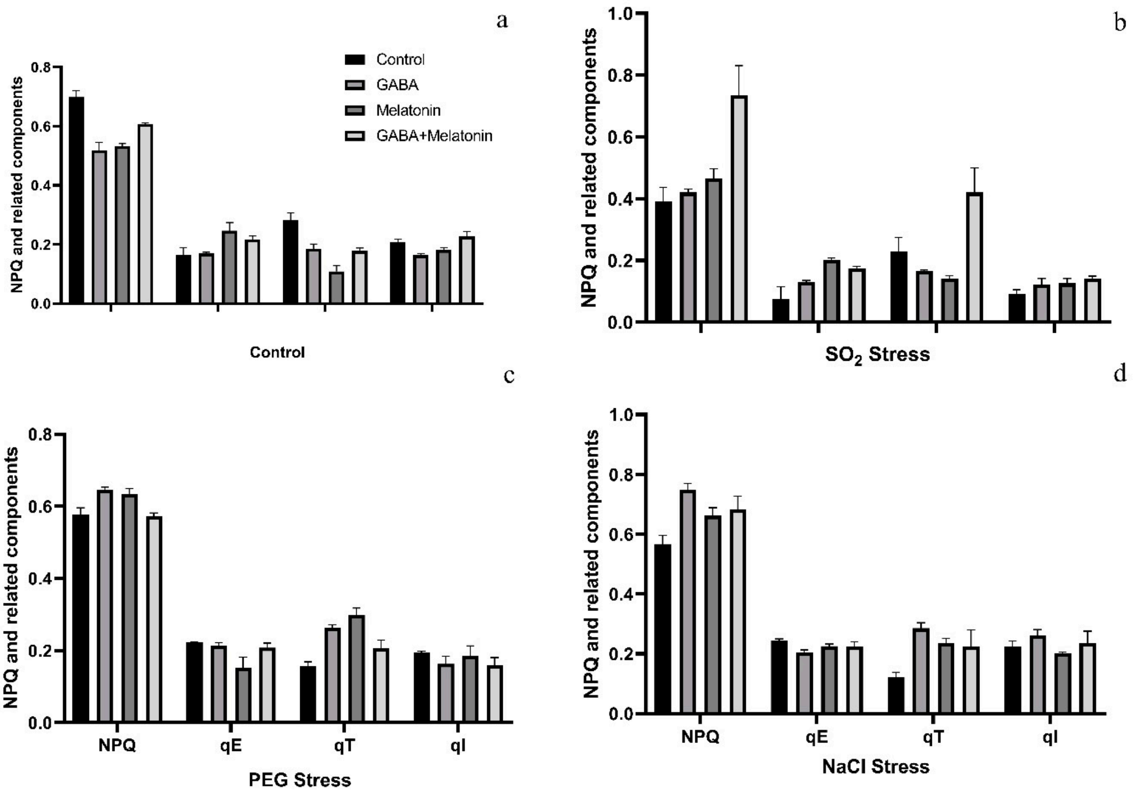

Supplementary Figure S3. NPQ and its components (qE, qT, qI) of *Vicia faba* plants primed with GABA (20 µM), melatonin (200 µM) and GABA (20 µM) + melatonin (200 µM) (a), when exposed to SO_2 stress (2 ppm) (b), PEG stress (-8 bar) (c) and NaCl stress (100 mM) (d).

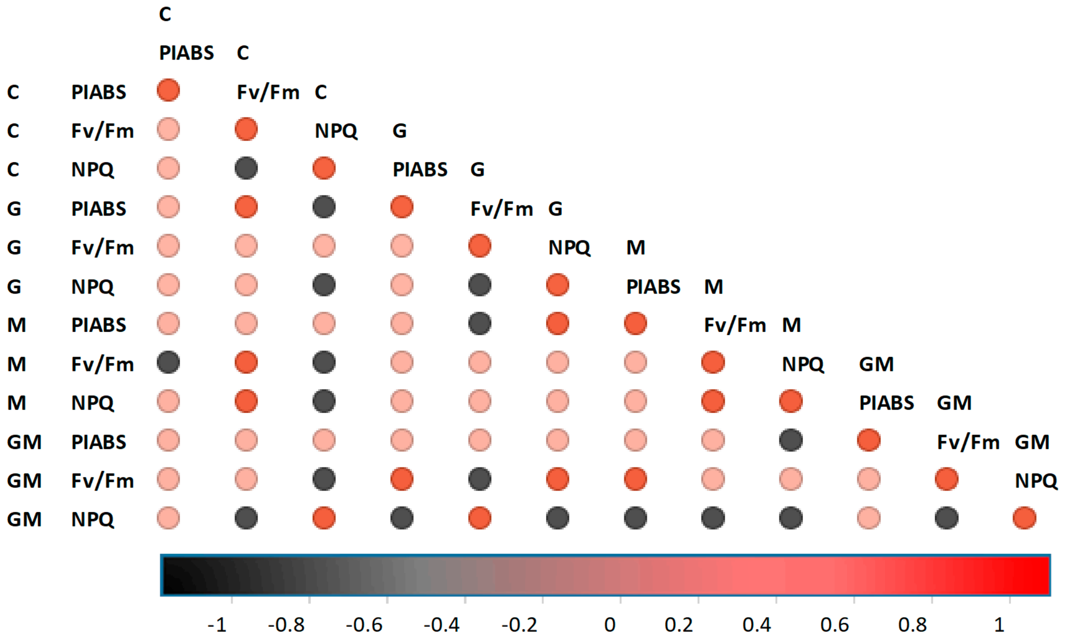

Supplementary Figure S4. Graphical representation of a correlation matrix of initial PI_{ABS} with F_V/F_M and NPQ in control and leaf discs of *Vicia faba* primed with Gamma-Aminobutyric Acid [(GABA (20 µM)], melatonin (200 µM) and GABA (20 µM) + melatonin (200 µM) . Red color represents positive correlation whereas black represents negative correlation. Color intensity is proportional to the correlation, which depicted in the legend at the bottom.

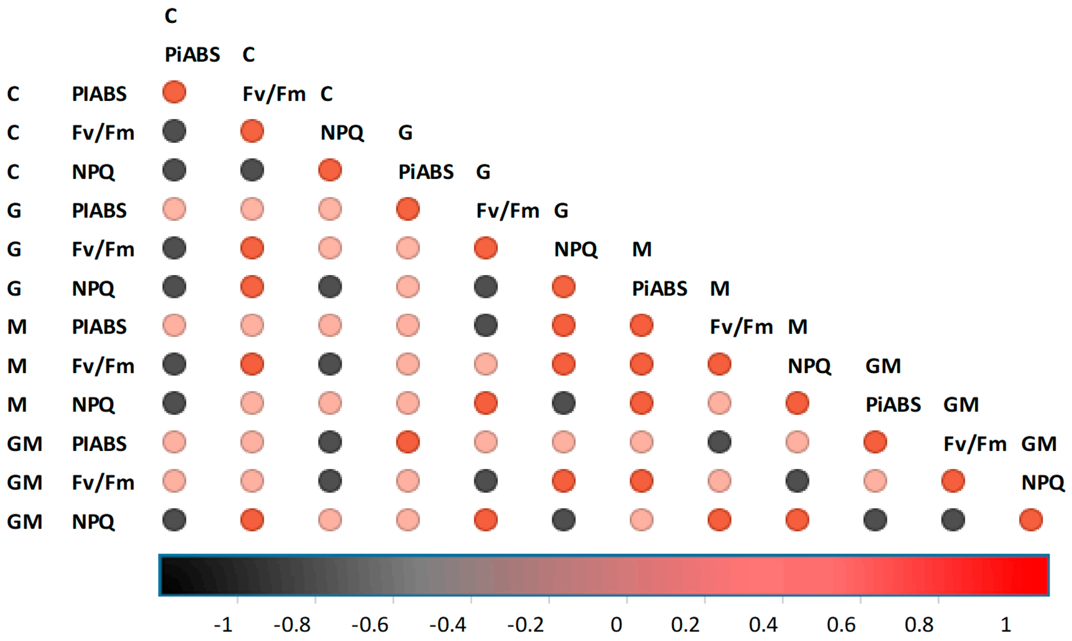

Supplementary Figure S5. Graphical representation of a correlation matrix of initial PI_{ABS} with F_V/F_M and NPQ in control and leaf discs of *Vicia faba* primed with Gamma-Aminobutyric Acid [(GABA (20 µM)], melatonin (200 µM) and GABA (20 µM) + melatonin (200 µM)when exposed to PEG stress. Red color represents positive correlation whereas black represents negative correlation. Color intensity is proportional to the correlation, which depicted in the legend at the bottom.

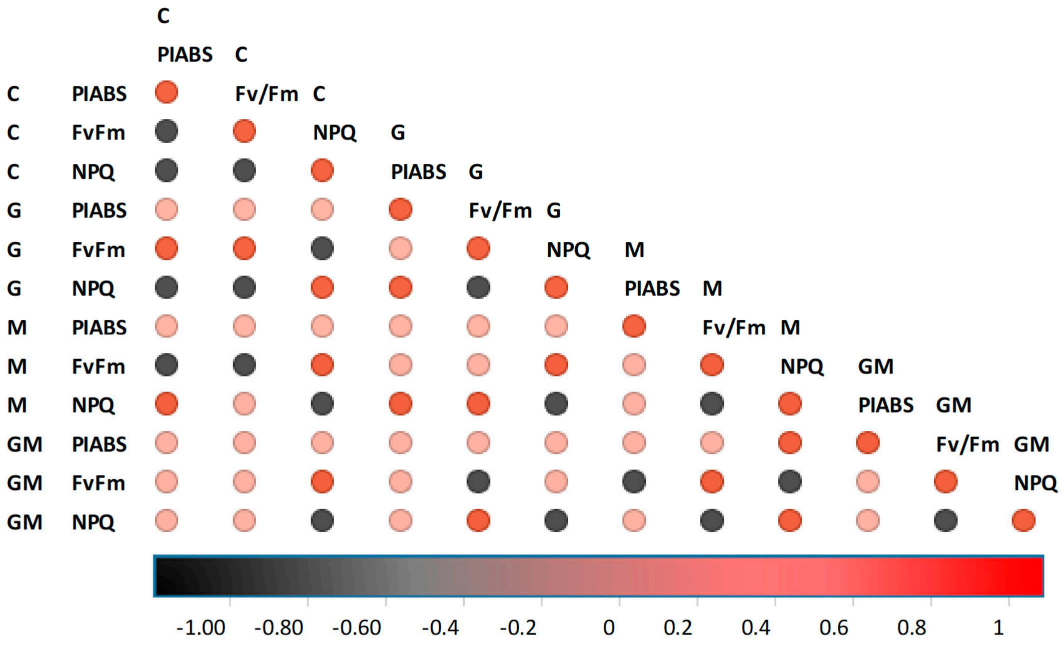

Supplementary Figure S6. Graphical representation of a correlation matrix of initial PI_{ABS} with F_V/F_M and NPQ in control and leaf discs of *Vicia faba* primed with Gamma-Aminobutyric Acid [(GABA (20 µM)], melatonin (200 µM) and GABA (20 µM) + melatonin (200 µM)when exposed to NaCl stress. Red color represents positive correlation whereas black represents negative correlation. Color intensity is proportional to the correlation, which depicted in the legend at the bottom.

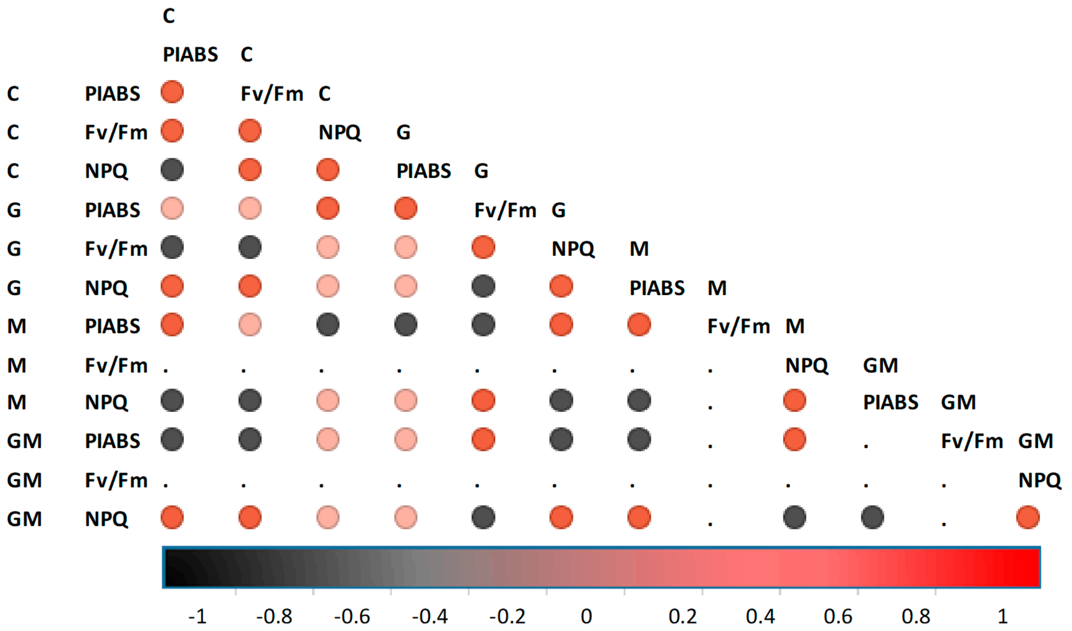

Supplementary Figure S7. Graphical representation of a correlation matrix of initial PI_{ABS} with F_V/F_M and NPQ in control and leaf discs of *Vicia faba* primed with Gamma-Aminobutyric Acid [(GABA (20 µM)], melatonin (200 µM) and GABA (20 µM) + melatonin (200 µM)when exposed to SO_2 stress. Red color represents positive correlation whereas black represents negative correlation. Color intensity is proportional to the correlation, which depicted in the legend at the bottom.

Kalaji MH (2017). Chlorophyll fluorescence: understanding crop performance—basics and applications (CRC Press).
